# From Waste to Resource: Driving Sustainability Through Eco-Friendly Substrates for Soilless Culture

**DOI:** 10.3390/plants15101459

**Published:** 2026-05-11

**Authors:** Wanlai Zhou, Wei Lin, Hong Wang

**Affiliations:** Institute of Urban Agriculture, Chinese Academy of Agricultural Sciences, Chengdu 610213, China; linwei01@caas.cn (W.L.); wanghong07@caas.cn (H.W.)

From high-tech greenhouses to urban vertical farms, the rapid global expansion of soilless cultivation has brought about unprecedented growing efficiency. However, this high productivity is heavily dependent on specific growing media—particularly peat and rockwool. Peat extraction destroys vital wetland carbon sinks, rockwool production is extremely energy-intensive, and its disposal also poses serious challenges. At the same time, agricultural and forestry activities generate large quantities of organic waste that are usually landfilled or incinerated, causing both resource waste and environmental burden. Converting waste into growing media could potentially address both issues. This Special Issue, entitled “Driving Sustainability: Innovations in Producing Eco-Friendly Substrates for Soilless Culture Systems”, aims to explore a circular alternative: transforming waste into high-performance growing media. After a rigorous review process, we have collected 12 original research and review articles that together map the potential, limitations, and future directions of this approach.

The papers collected here cover a wide diversity of feedstocks, processing methods, and target crops. Studies show that agricultural and forestry residues—pine bark, wood fiber, coffee silverskin, brewer’s spent grain, kenaf core, and green waste—as well as recycled construction waste, can serve as partial or complete substitutes for peat and perlite. Processing strategies include direct physical mixing, aging, composting, and an innovative ammonium incubation technique that rapidly eliminates phytotoxicity. Test crops include leafy vegetables (lettuce, lemon basil), a fruit vegetable (cucumber), ornamentals (sage), and woody shrubs (*Alyogyne huegelii* and *Goodenia ovata*). Thus, a unifying narrative emerges: organic and mineral waste-derived substrates can indeed drive sustainability, but their success is highly context-dependent, requiring species-specific optimization and careful management of two major bottlenecks—phytotoxicity and nitrogen immobilization.

## 1. Renewable Materials as Alternatives to Conventional Substrates: Feasibility Validation

Several studies evaluated locally available plant-derived materials as alternatives to conventional substrate components. In lemon basil, a mixture of 70% coconut fiber and 30% perlite significantly improved seed germination, seedling leaf number, and total leaf area [1]. In another trial, pine bark (8% *v*/*v*) was used to replace perlite in a coir-based substrate for lettuce. No negative effects on yield, chlorophyll content, or total phenolic content were observed; however, leaf iron and boron concentrations were elevated [2]. When wood fiber, coffee silverskin, and brewer’s spent grain were used to replace 10–40% of peat for two Salvia genotypes, the results showed that the optimal substrate formulation depends not only on the type and rate of the amendment but also, to a large extent, on the plant genotype [3]. Overall, the suitability of plant-derived materials as growing media depends on crop species, material processing, and management [3,4]. Future research should optimize pretreatment methods for different materials and investigate long-term stability in diverse cropping systems.

Sustainability in soilless systems is not limited to organic alternatives. One study explored the possibility of using pine bark-amended recycled construction waste (recycled sand, crushed concrete, crushed rock) as a capping substrate for urban plantings [5]. The pure mineral wastes were strongly alkaline (pH 9.2–12.3) and had poor water retention; however, after adding 50% pine bark, the pH reached acceptable ranges and supported the growth of both alkaline-tolerant and alkaline-sensitive species. The best-performing mix (recycled sand +10% pine bark) produced plant biomass comparable to that of scoria, a conventional quarried material. This opens the door to large-scale reuse of demolition waste in green infrastructure.

## 2. Tackling Phytotoxicity: From Composting to Ammonium Incubation

A primary obstacle for fresh green waste is its inherent phytotoxicity, mainly derived from phenolics, organic acids, and other allelopathic compounds. Several papers in this Special Issue mention phytotoxicity issues in plant-derived materials such as coffee silverskin and green waste [6,7]. One research article optimized an ammonium incubation process for green waste; using 1.5% ammonium carbonate at 30 °C for five days with daily aeration reduced total phenolic content by 74% and increased the germination index (GI) from 6.4% to 127%, effectively eliminating phytotoxicity [6]. When this treated green waste replaced 75% of peat in lettuce cultivation, biomass increased nearly five-fold compared to untreated waste, though it remained below the peat control due to residual physical limitations. This low-energy, low-cost method represents a breakthrough for rapid detoxification of urban green waste.

## 3. Nitrogen Immobilization: A Double-Edged Sword

High-carbon substrates, especially wood fiber and fresh woody residues, often induce strong nitrogen immobilization, leading to transient nitrogen deficiency. A study on cucumber grown in wood fiber–peat mixtures showed that when the wood fiber content was 50%, and an additional 13 g N per plant was supplied, the fruit number and fruit biomass per plant were significantly higher [8]. Another study used chlorophyll fluorescence kinetics to investigate the physiological impact of wood fiber-induced nitrogen immobilization on lettuce [9]. The results indicated that wood fiber substrates significantly reduced available nitrogen, decreased chlorophyll content by 14–36%, and impaired electron transfer on the donor side of photosystem II (OEC) and the acceptor side of photosystem I. Notably, this damage was partially reversible over time, suggesting that dynamic, stage-adjusted nitrogen management can mitigate negative effects. A comprehensive review framed nitrogen immobilization as a “double-edged sword”: short-term immobilization may cause crop stress, but the sequestered nitrogen, stored in microbial biomass and lignin-associated organic pools, forms a slow-release reservoir that reduces nitrate leaching [10].

## 4. Main Conclusions and Future Perspectives

Together, the 12 articles in this Special Issue provide compelling evidence that waste-derived materials can successfully replace peat, perlite, and rockwool in a wide range of soilless applications, provided that material-specific pretreatments (composting, ammonium incubation, aging, or blending) are applied and that nitrogen and water management are optimized according to crop growth stage. The two recurring challenges—phytotoxicity and nitrogen immobilization—are now better understood and can be addressed through the proposed technical and management frameworks.

Looking forward, we highlight two promising research directions that emerged from this collection but remain underexplored.

The first is the positive role of humic-like substances (e.g., fulvic acid) as biostimulants in substrate-based systems. A floating hydroponic trial with iceberg lettuce systematically evaluated the effects of fulvic acid (FA), amino acids (AA), and vermicompost (VC), alone and in combination. The treatment FA 40 ppm + VC 2 mL/L gave the highest total yield (17.15 kg/m^2^), a 17.94% increase over the control. Further analysis showed that the combined application of FA, AA, and VC not only significantly increased vitamin C, total phenolics, and flavonoid contents but also effectively reduced nitrate accumulation in leaves [11]. These findings highlight the important value of biostimulants in improving yield and nutritional quality. Future research should pay more attention to humic-like substances released during the degradation of organic waste-based substrates, systematically evaluating their potential as endogenous biostimulants and their long-term effects on crop growth.

The second future research direction is the great potential of urban green waste (pruned branches, fallen leaves) as a local renewable substrate resource. Green waste is widely distributed, produced in large quantities, and has the advantage of local supply. Being mainly composed of plant fibers, it can be converted—after proper treatment—into a substrate with good water-holding capacity and aeration, while also supplying essential nutrients. Nevertheless, green waste generally suffers from severe phytotoxicity and nitrogen immobilization. The ammonium incubation technique presented in this Special Issue offers a rapid, low-cost, and scalable treatment method. However, long-term studies under commercial greenhouse conditions are needed to validate its performance over multiple crop cycles and to integrate it with closed-loop nutrient management systems.

We call upon the horticultural research community, industry stakeholders, and policymakers to actively embrace these innovations. The transition from a linear to a circular soilless cultivation model is not only an environmental necessity but also an economic opportunity—reducing dependence on peat and rockwool and creating value from local by-products. The articles in this Special Issue provide a solid scientific foundation for this transition.

## Data Availability

No new data was generated for this article.
